# Family‐Centered Occupational Therapy Consultation for Children Under 18 Years Old: A Scoping Review

**DOI:** 10.1155/oti/1184326

**Published:** 2026-01-08

**Authors:** Marjan Shahbazi, Elaheh Hojati Abed, Samaneh Karamali Esmaili

**Affiliations:** ^1^ Department of Occupational Therapy, School of Rehabilitation Sciences, Iran University of Medical Sciences, Tehran, Iran, iums.ac.ir

**Keywords:** consultation, family-centered care, occupational therapy, parent-mediated intervention, pediatrics

## Abstract

**Introduction:**

Family‐centered occupational therapy provided in children′s natural environments can enhance learning, development, participation, and caregiver competence. Consultation is a key approach within this model. This scoping review examined the literature on family‐centered occupational therapy consultation for individuals under 18 years old and its reported effects on families and goal achievement.

**Method:**

Following PRISMA‐ScR guidelines, five databases and gray literature were searched (2000–April 2025). Studies were included if they described synchronous consultation between families and occupational therapists. Data were extracted on study design, intervention characteristics, and outcomes.

**Results:**

Then, 15 studies met inclusion criteria (12 quantitative and three qualitative). Of the quantitative studies, eight reported statistically significant improvements in child or parent outcomes (e.g., occupational performance, participation, and parental confidence), three reported mixed or nonsignificant results, and one was descriptive only. Qualitative studies consistently reported increased caregiver understanding, confidence, and ability to implement strategies in natural environments. Most interventions occurred in schools or home programs, with limited evidence from telehealth, adolescents, or non‐Western contexts.

**Conclusion:**

Family‐centered consultation in pediatric occupational therapy shows promise for improving participation‐related outcomes and caregiver competence but remains methodologically heterogeneous and understudied in certain populations and formats. Further research should address these gaps to strengthen the evidence base.

## 1. Introduction

Recent investigations have shown that pediatric intervention methods targeting the natural environment of the child and family lead to greater learning, development, competence, and occupational participation [1, 2]. This finding can be justified as follows: Caregivers or family members offer unique perspectives on a child′s performance because they engage with the child in various settings and activities that involve different demands and levels of support [3]. This encourages discussion and collaboration between families and professionals to identify strategies that enhance the child′s participation in various contexts, such as home, school, and community. This approach aligns with the principle of family‐centered practice [4, 5].

Additionally, this aligns with the principles of contemporary models in occupational therapy, such as the model of human occupation (MOHO) [6], the Canadian Model of Occupational Performance (CMOP) [7], and the ecology of human performance (EHP) model [8]. In the models discussed, there is a strong emphasis on utilizing occupational goals and implementing interventions that take into account the client′s context and environment as integral components of the treatment process [9, 10].

Consultation is a family‐centered intervention [11] that incorporates elements of both education and advocacy. It follows a four‐step process: Reflecting on the situation, preparing for action, implementing that action, and ensuring positive outcomes are sustained over time. The primary goal of consultation is to empower individuals to help themselves. In essence, consulting involves supporting a client, agency, or other service provider in recognizing and analyzing issues, delivering relevant information and advice, and formulating actionable strategies [12].

Many people often confuse consultation with counseling and coaching [13]. Coaching primarily focuses on the future, emphasizing goal setting and finding solutions, whereas consulting is more oriented towards addressing present and past issues. It involves examining what went wrong to identify the root causes of a problem and seeks to address or resolve it [14]. Counseling, in its various forms—including play therapy, art therapy, and psychodynamic therapy—cultivates a distinctive therapeutic relationship that connects with a child′s world. This relationship is characterized by being safe, authentic, confidential, and nonintrusive, yet purposeful in observing aspects such as the child′s general appearance, behavior, mood/affect, cognition, motor skills, play, speech/language, and interactions with the therapist. The goal is to facilitate active behavior modification by exploring behavioral options and practicing new behaviors [15].

In contrast, pediatric occupational therapy consultations involve not only parents but also assistants, childcare providers, and other adults who spend significant time with the child [16]. This collaborative approach enables caregivers to develop skills that can be generalized to the child′s natural environment and tailored for long‐term use, while also helping them adapt activities to facilitate the child′s participation. Occupational therapists play a crucial role by enhancing parents′ understanding of the physiological and health‐related issues that influence their child′s behavior and by assisting them in implementing strategies that support everyday performance across various contexts [17].

In clinical practice, such consultations are often conducted in natural settings like homes, schools, and community centers, where therapists collaborate directly with caregivers to design and modify activities that enhance children′s participation [16]. For instance, recent studies have shown that parent‐implemented home programs guided by occupational therapists can improve children′s fine motor and self‐care skills, while simultaneously increasing parental confidence and reducing stress [18, 19]. These real‐world applications highlight the practical relevance of consultation beyond theoretical models.

Although family‐centered practice has been increasingly emphasized in pediatric occupational therapy, there remains a lack of clarity about how consultative interventions are implemented across different settings, age groups, and diagnostic categories [20, 21]. Existing literature mostly outlines general principles of family‐centered care but does not systematically map how consultation is operationalized, its outcomes, and its effectiveness [5, 11]. Because occupational therapy is inherently concerned with participation and occupational performance [22], this review intentionally focused on studies addressing children′s occupational needs and goals rather than impairment‐based outcomes, to provide a practice‐relevant synthesis for clinicians and researchers. Accordingly, the aim of this scoping review is not to evaluate the effectiveness of consultation but to systematically identify, chart, and synthesize the available evidence on occupational therapy consultation with families of children under 18, describing the range, characteristics, and reported outcomes of these interventions to highlight current practices and gaps for future research.

## 2. Method

The scoping review based on the framework outlined by Levac et al. [23] is an ideal approach for surveying the available evidence and assessing the extent of existing studies while also identifying knowledge gaps. Choosing this methodology allows for a broader exploration, description, and understanding of current research. The Preferred Reporting Items for Systematic Reviews and Meta‐Analyses extension for scoping reviews (PRISMA‐ScR) checklist [24] was employed to enhance reporting and provide a clear visualization of the article selection process (File S1).

### 2.1. Research Question

The research question for this scoping review was formulated using a modified PICO approach:

“What is known from the literature about the characteristics, implementation processes, and reported outcomes (outcome) of family‐centered occupational therapy consultation (Intervention) for children under 18 years old and their families (population)?”

Consistent with the Joanna Briggs Institute (JBI) framework [25], the question can also be expressed using the PCC (population–concept–context) format:

“What is known from the literature about family‐centered occupational therapy consultation (concept) for children under 18 years old (population) across clinical, educational, and community contexts (context)?”

### 2.2. Search Strategy

To ensure a thorough search, the first author, under the supervision of two faculty members responsible for teaching the course on collaborative consulting in occupational therapy, conducted a search for eligible articles in the following online databases. The databases searched included PubMed, Web of Science, Scopus, PsycINFO, and OTseeker covering the period from January 1, 2000, to April 2025 to capture the most recent research information. Additionally, Google and Google Scholar were utilized to search for gray literature.

The primary reason for restricting the search time span to this range is that consultation has become more prominent in occupational therapy since 2000. This change was marked by the legitimization of occupational therapists as educational practitioners in the inclusive education sector through the Special Education 2000 initiative, rather than being viewed solely as health‐based practitioners [26].

The search terms focused on three main concepts: (1) family‐centered care, (2) collaborative consulting, and (3) occupational therapy. The detailed PubMed search strategy, including all search terms and limits applied, is provided in File S2.

### 2.3. Study Selection Criteria

Inclusion and exclusion criteria were established a priori to guide the literature search and address the research question [23, 24]. Specific criteria are summarized in Table 1. The first author independently screened the titles and abstracts identified through the search, with supervision from the two co‐authors. The full texts of potentially eligible studies were then evaluated against these criteria. Any disagreements regarding eligibility were resolved through discussion until a consensus was reached among all authors. Due to the limited number of included studies and the exploratory nature of the review, formal interrater reliability statistics (e.g., Cohen′s kappa) were not calculated; however, agreement was reached through iterative discussions at each stage of the screening process [24, 27].

**Table 1:** Inclusion and exclusion criteria for study selection.

**Framework**	**Inclusion criteria**	**Exclusion criteria**
Population (P/P in PCC)	Children and adolescents from birth to 18 years old and their parents/caregivers involved in occupational therapy consultation.	Studies including adults, populations over 18 years, or interventions directed only at parents without child involvement.
Intervention (I in PICO/C = concept in PCC)	Family‐centered occupational therapy consultation (individual or group), delivered face‐to‐face, online, or hybrid. Both direct + consultative and purely consultative models included.	Studies focusing only on counseling, coaching, or therapy without consultation; interventions not involving occupational therapy.
Comparison (C in PICO)	Not applicable (scoping review design, no control group required).	—
Outcome (O in PICO)	Occupational performance, participation, goal attainment, family‐related outcomes (e.g., parental competence, satisfaction).	Studies reporting only impairment/body function outcomes (e.g., range of motion, strength) without occupational performance measures.
Context (C in PCC)	Clinical, educational, home, and community settings where OT consultation occurred with active involvement of parents/caregivers (in addition to teachers or other providers, if applicable).	Studies conducted in schools where consultation was only with teachers and parents were not involved; studies in acute/critical care units; contexts outside pediatrics; or non‐OT professional consultations.
Study design	Peer‐reviewed quantitative (RCT, quasi‐experimental, pre–post, before–after) and qualitative studies; published in English between 2000–2025.	Non–peer‐reviewed reports, conference abstracts, dissertations, studies published before 2000, or in languages other than English.

### 2.4. Critical Appraisal

To assess the methodological quality of the included studies, we employed standardized appraisal tools tailored to each study′s design. For quantitative studies, we utilized the McMaster University Critical Review Form for Quantitative Studies [28], while qualitative studies were evaluated using the McMaster Critical Review Form for Qualitative Studies [29]. The levels of evidence for quantitative research were classified according to the criteria established by the Centre for Evidence‐Based Medicine, as outlined by Tomlin and Borgetto [30]. This approach ensured a consistent and transparent evaluation of study quality across various designs. The outcomes of this appraisal, including quality ratings and levels of evidence for each included study, are summarized in Table 2 and detailed in the Results section.

**Table 2:** Critical appraisal of included studies.

**Citation**	**Country**	**Design/level of evidence**	**Sample**	**Intervention specifics**	**Outcome measures**	**Fidelity reporting**	**Bias/confounders**	**Overall quality**	**Main limitation/note**
King et al. 2000 [31]	Canada	One‐group pretest–posttest (Level III)	50 children	School‐based OT, family‐centered functional model	GAS, AAPS, VABS, SFA, CSQ	Partial	No control group	Moderate	Feasibility only
Cohn et al., 2000 [32]	USA	Qualitative (Level IV)	17 parents	Sensory integration consultation	Interviews	N/A	Small sample	Moderate–good	No measurable outcomes
Dreiling & Bundy, 2003 [33]	USA	Quasi‐experimental between‐group (Level II)	20 preschoolers	Consultative vs direct–indirect OT	GAS	Not clear	Small, not randomized	Moderate–weak	Limited fidelity
Law et al., 2005 [34]	Canada	Prospective before–after (Level III)	167 children	Home & community OT, direct + consultative	COPM, PedsQL, MPOC	Partial	No control, concurrent therapy	Moderate	Limited fidelity
Bayona et al., 2006 [35]	Canada	One‐group pretest–posttest (Level III)	23 children	Consultative OT, school‐based	VABS, SFA, CSF	Partial	Small sample	Moderate‐Weak	Limited generalization
Schaaf & Nightlinger, 2007 [36]	USA	Case study (Level IV)	1 child	Direct SI therapy, some consultation	SP, GAS, interviews	Case reported	Single child, parent report	Weak	Not generalizable
Cason, 2009 [37]	USA	Pilot telerehabilitation (Level III)	2 families	Remote OT consultation	Satisfaction, cost	Limited	Very small sample	Weak	Pilot only
Ratzon et al., 2010 [38]	Israel	Quasi‐experimental, 2 groups (Level II)	45 children	Classroom + home program	VMI, DTVP‐2, SFA	Partial	Cointerventions, unclear adherence	Moderate	Mixed results
Gibbs & Toth‐Cohen, 2011 [39]	USA	Pilot study (Level III)	4 families	Family‐centered telerehab	SPM‐Home	Limited	Very small sample	Weak	Feasibility only
Missiuna et al., 2012 [40]	Canada	Mixed‐methods feasibility (Level III)	428 students, 8 OTs	Partnering for change	Surveys, interviews	Good for process	No control group	Good for feasibility	Needs RCT for effect
Wuang et al., 2013 [18]	Taiwan	RCT (Level II)	64 children	Parent‐delivered OT home program	COPM, BOT‐2, CAPE	Partial	Blinding unclear	Good	Significant gains
Solomon et al., 2014 [19]	USA	RCT (Level II)	128 children	PLAY Project home consultation	ADOS‐G, COPM, PSI	Fidelity monitored	Blinding unclear	Good	Strongest evidence
Mische Lawson et al., 2023 [41]	USA	Pretest–posttest (Level III)	17 children	Sensory garments for ASD	COPM, GAS, PSI‐SF, PSOC	Partial	Small sample	Moderate	No control
Creen et al., 2023 [42]	Australia	Qualitative thematic (Level IV)	38 parents, 12 children	Single‐session therapy	Interviews	N/A	Self‐selection bias	Good	Limited generalization
Grandisson et al., 2025 [43]	Canada	Mixed‐methods evaluation (Level III)	7 families	Capacity‐building OT service	COPM, MPOC	Limited	Small sample	Moderate‐Good	Encouraging, limited data

### 2.5. Data Extraction

The researcher developed a form to extract data from the retained articles. Three categories of data were collected, including methodology, intervention specifications, and outcome measures, following the identification of family‐oriented occupational therapy consultation for children under 18 years old in the included studies.

### 2.6. Data Verification and Supervision

Data extraction was undertaken by the first author and closely supervised by the two senior authors to ensure methodological rigor and accuracy. The supervisory role was operationally defined as an active quality control process that included (a) reviewing the data extraction form prior to use to confirm its comprehensiveness and alignment with the study objectives, (b) independently verifying the accuracy and completeness of approximately 30% of randomly selected extracted data against the original publications, and (c) resolving any inconsistencies or ambiguities through team discussion until full consensus was achieved.

Regular supervisory meetings were held throughout the data extraction phase to monitor progress, address uncertainties, and maintain consistency across the extracted variables. Although this approach provided continuous methodological oversight, it did not involve two fully independent data extractors as recommended in best‐practice guidelines. This limitation and its potential implications for bias are further discussed in the Limitations section.

## 3. Results

**Figure 1:**
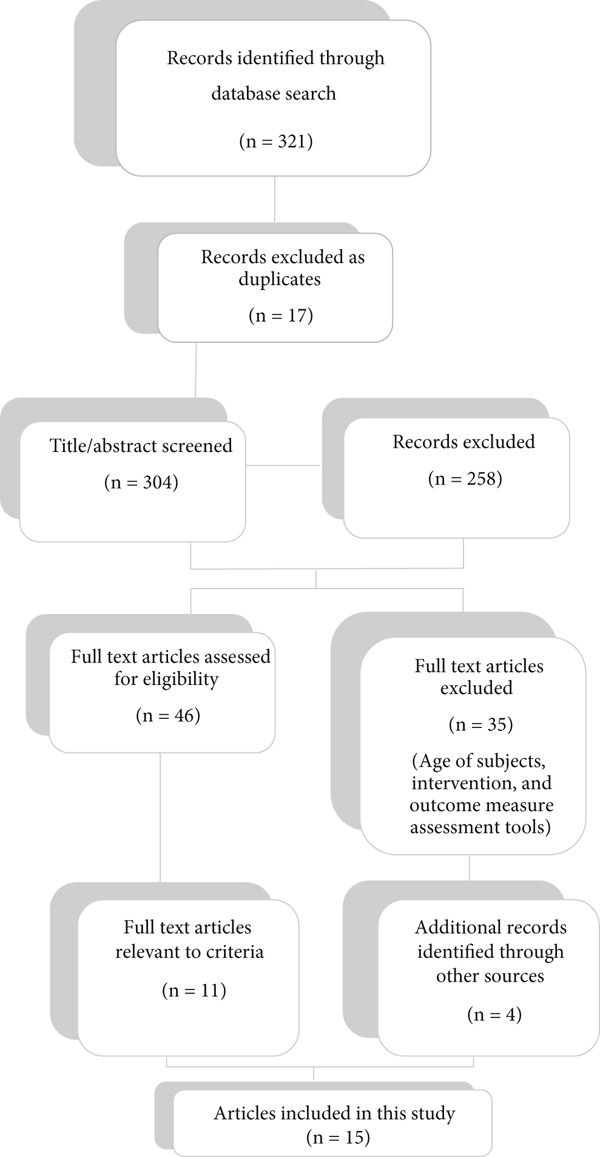
Flowchart of search strategy and study selection.

This scoping review was aimed at exploring current consulting interventions in occupational therapy services for families with children under 18 years old. A total of 321 articles were initially retrieved, with 304 remaining after duplicates were removed. Screening titles and abstracts led to the exclusion of 258 articles due to irrelevance, resulting in 46 full‐text articles reviewed. After applying inclusion and exclusion criteria, 11 articles were retained, and a background search of references identified four additional eligible articles, yielding a final sample of 15 studies included in the review (see Figure 1).

**Figure 2:**
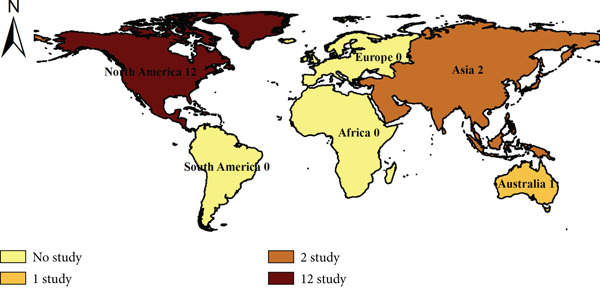
Geographic distribution of included studies by continents.

The included studies were primarily conducted in North America, particularly Canada [31, 34, 35, 40, 43] and the United States [19, 32, 33, 36, 37, 39, 41], with some from Australia [42], Israel [38], and Taiwan [18] (see Figure 2). This indicates that most evidence comes from Western, high‐income settings, with limited representation from Eastern or low‐ and middle‐income countries, where cultural and service contexts may differ.

All included studies underwent critical appraisal for methodological quality and level of evidence using standardized tools, summarized in Table 2. Two randomized controlled trials were classified as Level II evidence [18, 19], while most other quantitative studies were rated as Level III evidence due to quasiexperimental or prospective designs [31, 34, 35, 41]. Three qualitative studies showed moderate‐to‐good methodological rigor [32, 42, 43]. Reporting on intervention fidelity, participant adherence, and potential confounders varied considerably, reflecting the research′s heterogeneity and early stage. This appraisal indicates that the evidence for occupational therapy consultation with families of children under 18 is growing but remains methodologically diverse and primarily at lower levels of evidence.

These assessments provide context for interpreting the review′s findings, organized into three main domains: study designs and populations, characteristics of consultation interventions, and outcome measures and reported effects (see Table 3). Additionally, two supporting sections summarize (a) overall outcome patterns across studies (e.g., number reporting significant improvements vs. no differences) and (b) key gaps identified by diagnostic group, age category, and intervention format. This structure aids in achieving a comprehensive understanding of the current evidence base and its limitations.

**Table 3:** Summary of studies included in the review.

**Citation**	**Country**	**Methodology**	**Sample**	**Intervention specifications**	**Outcome measure**	**Findings**	**Level of evidence**
King et al., 2000 [31]	Canada	Quasi‐experimental (one‐group pretest–posttest)	50 children (ages 5–12)	School‐based, family‐centered, functional approach	GAS, AAPS, VABS, SFA, CSQ	Significant improvements in communication, productivity, mobility	Level III
Cohn et al., 2000 [32]	USA	Qualitative (grounded theory)	17 parents	Sensory integration approach, parental perspectives	SIPT, SP, CBC	Parents reported improvements in social participation, competence	Level IV
Dreiling & Bundy, 2003 [33]	USA	Quasiexperimental between‐group	20 children (ages 3–5)	Consultative vs direct–indirect model	GAS	No significant differences	Level II
Law et al., 2005 [34]	Canada	Prospective before–after study	167 (1) children/youth	Community and home‐based OT, consultative	COPM, PedsQL, MPOC	Significant improvement in occupational performance, satisfaction	Level III
Bayona et al., 2006 [35]	Canada	One‐group pretest–posttest	23 children (ages 5–8)	School‐based consultative OT	VABS, SFA, CSF	Significant improvements in fine motor and school‐related tasks	Level III
Schaaf & Nightlinger, 2007 [36]	USA	Case study	1 child (4.5y)	Sensory integrative OT, some consultation	SP, GAS, interview	Improvements in performance and participation	Level IV
Cason, 2009 [37]	USA	Pilot program evaluation	2 families	Telerehabilitation consultative OT	Satisfaction, cost	Families satisfied, perceived benefit of telerehab	Level III
Ratzon et al., 2010 [38]	Israel	Prospective two‐group study	45 children	Classroom consultation + home parental program	VMI, DTVP‐2, SFA	No significant improvement in visual–motor skills	Level II
Gibbs & Toth‐Cohen, 2011 [39]	USA	Pilot project	4 families	Family‐centered OT via telerehabilitation	SPM‐Home	Enhanced parental engagement	Level III
Missiuna et al., 2012 [40]	Canada	Mixed‐methods feasibility	428 students, 8 OTs	Partnering for change (school‐based capacity building)	Surveys, interviews, logs	Improved teacher knowledge, parent empowerment	Level III
Wuang et al., 2013 [18]	Taiwan	Randomized controlled trial (RCT)	64 children	Parent‐delivered OT home program	COPM, BOT‐2, CAPE	Significant gains in fine motor skills, participation	Level II
Solomon et al., 2014 [19]	USA	RCT	128 children	PLAY Project home consultation	ADOS‐G, MBRS, COPM, PSI	Positive changes in parent–child interaction	Level II
Mische Lawson et al., 2023 [41]	USA	Pretest–posttest repeated measures	17 children	Sensory garment intervention for ASD	COPM, GAS, PSI‐SF, PSOC	Significant improvements in COPM, GAS; no change in stress	Level III
Creen et al., 2023 [42]	Australia	Qualitative study	38 parents, 12 children	Single‐session therapy consultation	Interviews	Parents reported increased understanding, confidence	Level IV
Grandisson et al., 2025 [43]	Canada	Mixed‐methods evaluation	7 families	Capacity‐building OT service	COPM, MPOC, interviews	Parents reported improved confidence, routines	Level III

Abbreviations: AAPS, Articulation Proficiency Scale; ADOS‐G, Autism Diagnostic Observation Schedule; BOT, Bruininks–Oseretsky Test of Motor Proficiency; CAPE, Children′s Assessment of Participation and Enjoyment; CBRS, Child Behavior Rating Scale; CES‐D, Center for Epidemiologic Studies Depression Scale; COPM, Canadian Occupational Performance Measure; CSP2, Child Sensory Profile‐2; CSQ, Client Satisfaction Questionnaire; DTVP‐2, developmental test of visual perception; GAS, goal attainment scaling; MBRS, Maternal Behavior Rating Scale; MCDI, MacArthur Communicative Development Inventory; MPOC, measure of processes of care; PedsQL, Pediatric Quality of Life Inventory; PSOC, Parent Sense of Competence Scale; PSI, Parenting Stress Index; PSI‐SF, Parent Stress Index‐Short Form; SCQ, Social Communication Questionnaire; SFA, School Function Assessment; SRS‐2, Social Responsiveness Scale‐2; SSICQ, Satisfaction with School‐Based Intervention and Communication Questionnaire; VABS, Vineland Adaptive Behavior Scale; VMI, visual motor integration.

### 3.1. Study Designs and Populations

Then, 15 studies met the inclusion criteria for this scoping review. Among these, 12 were quantitative studies that utilized various designs—including between‐group, single‐group pre–post, quasiexperimental, and prospective approaches—to examine consulting interventions for families of children and adolescents with a range of disabilities such as autism spectrum disorder (ASD), motor delays, cerebral palsy, attention‐deficit hyperactivity disorder (ADHD), anxiety, speech and language disorders, specific learning disabilities, intellectual disabilities, and developmental coordination disorder (DCD) [18, 19, 31, 33–41]. While these designs provide valuable preliminary evidence, they are limited in their ability to control for internal validity threats such as maturation and individual differences. Additionally, three qualitative studies investigated parent experiences and perceptions of their involvement in these interventions, offering rich insights into the contextual and experiential aspects of consultation [32, 42, 43]. A summary of the individual results and main findings from each included study is presented in Table 3.

### 3.2. Characteristics of Consultation Interventions

Across the included studies, consultation interventions were predominantly delivered in naturalistic contexts such as homes, schools, and community centers, allowing strategies to be embedded into children′s daily routines and family priorities [18, 19, 31, 33–35]. Most interventions combined structured caregiver training, observation of the child′s performance, collaborative problem‐solving, and guided practice with therapist feedback to enhance parents′ competence and children′s participation [18, 32, 33, 42, 43]. Nine studies described occupational therapy–specific consultation, while six examined multidisciplinary models integrating occupational therapy with other services, reflecting the adaptability of consultation to different service systems [18, 31–33, 38, 39, 41–43]. Although the duration and intensity of programs varied from single‐session consultations to multimonth follow‐ups, a consistent emphasis was placed on tailoring recommendations to family needs and empowering caregivers to implement strategies in natural environments [18, 31, 34, 35, 42]. Only a few studies explored telehealth or hybrid delivery formats, but these demonstrated feasibility for extending services to underserved families [37, 39]. Taken together, these features highlight consultation as a flexible, family‐centered model that strengthens caregiver capacity while remaining heterogeneous in structure, content, and delivery methods across studies.

### 3.3. Outcome Measures and Reported Effects

While several qualitative studies primarily focused on parents′ perceptions and outcomes related to parenting (e.g., [42, 43], most quantitative studies examined both child and parent outcomes [19, 31, 33, 38, 41]. Widely used standardized tools, such as Goal Attainment Scaling (GAS) and the Canadian Occupational Performance Measure (COPM), were employed to assess occupational performance and participation. Recent studies have shown statistically significant improvements in children′s occupational performance and participation outcomes following consulting interventions, as measured by GAS and COPM [41, 43]. These assessments are psychometrically robust, client‐centered, individualized, and validated for populations under 18 years of age [44]. Such outcome measurements align with family‐centered practices and interventions that target family occupations and participation [45, 46].

#### 3.3.1. Overall Patterns of Outcomes Across Studies

Across the 12 quantitative studies included in this review, eight reported statistically significant improvements in at least one child or parent outcome following consultation (e.g., occupational performance, participation, or parental confidence) [18, 19, 31, 34–37, 39], three reported mixed or nonsignificant effects [33, 38, 41], and one primarily provided descriptive data without testing for change [40]. In contrast, the three qualitative studies consistently described parents′ perceptions of increased understanding, confidence, and ability to implement strategies in natural environments [32, 42, 43]. Taken together, these findings indicate that while consultation interventions show promise for improving participation‐related outcomes, they remain methodologically heterogeneous and vary in effect size and reporting rigor (see Table 2).

#### 3.3.2. Identified Gaps

Several important gaps emerged from this scoping review. Most included studies focused on children with ASD or mixed developmental diagnoses [19, 39, 41], leaving limited evidence for other conditions such as cerebral palsy, ADHD, or speech and language disorders. In addition, very few studies examined consultation for infants or toddlers, and only a small number addressed adolescents or youth transitioning to adulthood [34, 42]—a critical developmental period when occupational roles, school‐to‐work transitions, and independence skills become increasingly salient. Finally, although home‐ and school‐based consultation was most common [18, 31, 35], there was a lack of research on telehealth, group‐based, or hybrid consultation formats. These gaps underscore the need for more diverse and rigorous research across diagnostic groups, age categories, and delivery methods, particularly studies that examine consultation approaches tailored to the unique needs of adolescents and their families during transitional stages.

## 4. Discussion

### 4.1. Efficacy of Consultation

This scoping review provides a comprehensive overview of how occupational therapy consultation with families of children under 18 is described and evaluated across diverse contexts. Although many included studies reported positive outcomes of consultation on participation and occupational performance [19, 34, 36], several showed mixed or nonsignificant effects [33, 38]. These inconsistencies may reflect differences in study design, small sample sizes, heterogeneity of participant diagnoses, variability in intervention fidelity, and the influence of contextual factors such as service delivery models and cultural norms. Together, these findings highlight both the potential and the limitations of the current evidence base and provide a foundation for examining how consultation practices may vary across cultural and systemic settings.

The geographical and cultural distribution of the included studies reveals a marked imbalance between Western, high‐income contexts and Eastern or non‐Western settings. Evidence from Jordan and Iran underscores how cultural norms and systemic factors shape occupational therapy practice in ways that differ from Western models. Malkawi et al. [47] described how occupational therapy practice in Jordan is strongly influenced by cultural norms that affect practitioner–family interactions and service delivery. Similarly, Khayatzadeh‐Mahani et al. [48] found that Iranian occupational therapists often experience a “declination from occupation‐based practice,” reflecting pressures from healthcare systems and societal expectations that lead to more therapist‐directed and less client‐centered approaches.

These cultural patterns suggest that consultation, as conceptualized in Western literature, may be implemented and experienced differently in Eastern contexts, where family engagement, decision‐making, and expectations of the therapist′s role can diverge from client‐centered models. Consequently, while the findings of this review provide valuable insights into current practices, they cannot yet be assumed to represent the global diversity of occupational therapy consultation. Future research should directly examine consultation practices across diverse cultural settings—including adolescents and transitional stages—to clarify potential differences and inform culturally responsive, family‐centered approaches.

### 4.2. Parental Experiences

This evidence suggests that consulting with caregivers positively impacts parental self‐efficacy [19, 31, 42]. However, consulting is not always intuitive, and many therapists are unsure how to effectively guide caregiver engagement [26]. For this reason, service providers in this area require ongoing training to implement interventions that are both culturally responsive and competent [21].

Studies varied in their use of consulting strategies. Practitioners employed relatively few strategies in their practice, including observation, conversation, demonstration, and practice with feedback [38, 41]. However, when implementing a consulting model, it is essential for practitioners to enhance parents′ sense of competence by strengthening their ability to communicate effectively with their children, thereby fostering development within a natural environment [21]. Notably, to underscore the importance of integrating interventions into real family contexts, only one study utilized play as the primary activity for the intervention [19]. This type of activity necessitated strategies that ensured higher levels of active caregiver participation, such as caregiver practice with feedback. Consequently, the implementation of these strategies underscored the significance of provider training.

Furthermore, the three qualitative studies provide valuable insight into the contextual and experiential dimensions of consulting interventions, which is especially important given the limited attention to adolescents and transitional stages in the current literature [42]. These studies demonstrate that consultation has the potential to positively impact families not only during early intervention years [32, 43] but also at other critical times and transitions in children′s and families′ lives. This aligns with occupational therapy′s life course perspective, which advocates for interventions that support participation and family functioning across developmental stages [49]. Collectively, these findings reinforce that family‐centered consultation can produce positive outcomes for both children and their families without relying exclusively on traditional, child‐centered intervention techniques.

### 4.3. Implications for Practice and Training

The findings of this review have several implications for occupational therapy practice. The review question focused on how family‐centered consultation supports children′s occupational performance and parental outcomes, and the results demonstrate that consultation can both enhance children′s participation and strengthen caregivers′ sense of competence. By aligning with these objectives, the review emphasizes consultation as a viable alternative or complement to direct intervention.

From a clinical perspective, practitioners should incorporate structured caregiver training, guided practice with feedback, and collaborative goal‐setting into consultation models. These strategies have been linked to stronger improvements in occupational performance and parental self‐efficacy. Additionally, therapists are encouraged to embed interventions into natural environments (home, school, and community), use play and daily routines as vehicles for learning, and adapt consultation approaches to cultural contexts. Such recommendations ensure that interventions are both effective and sustainable in real‐life family settings.

In terms of relevance to key groups, parents benefit from improved self‐efficacy and advocacy skills, therapists gain effective tools for family engagement, researchers can build on identified gaps (such as adolescents and underrepresented diagnostic groups), and policymakers can consider consultation as a cost‐effective service delivery model that extends therapist impact without relying solely on direct treatment. Collectively, these implications strengthen the relevance of the review′s findings for multiple stakeholders.

Research on consultation indicates that this service delivery model is as effective as direct intervention. It enhances parents′ skills, modifies various aspects of the “human environment”—such as attitudes, beliefs, and interactions—to optimize a client′s performance and fosters collaboration and communication [33]. Additionally, incorporating consultation as part of treatment to engage parents and gather information from them proves to be effective as well [41].

However, the evidence base remains somewhat inconsistent. Eight out of the 12 quantitative studies indicated that consultation led to significant improvements in goal attainment and occupational performance among pediatric clients, with additional positive effects observed in parents′ sense of self‐competence and efficacy [19, 31, 39, 41]. However, several studies, including those by Dreiling and Bundy [33] and Ratzon et al. [38], did not demonstrate significant differences compared with other treatment methods. Across studies, factors such as varying levels of parental involvement in the intervention and differences in children′s cognitive and linguistic abilities appeared to influence the successful implementation and outcomes of consultation [19, 38].

A closer examination of the mixed findings across the 15 included studies indicates that several contextual and methodological factors likely contributed to the variability in reported outcomes. For example, levels of parental engagement differed markedly: interventions incorporating structured caregiver training, opportunities for guided practice, and ongoing feedback (e.g., [19, 43] tended to show stronger gains in children′s occupational performance and parental self‐efficacy than those offering only brief consultation or written materials [33]. The duration and intensity of consultation also varied widely, ranging from single‐session models to multimonth programs, which likely influenced both the depth of skill acquisition and the sustainability of outcomes. Moreover, the considerable heterogeneity of participating children—including differences in diagnosis, developmental level, and communication abilities—may have attenuated effect sizes in some studies and limited comparability across trials. Taken together, these factors suggest that future research should not only evaluate the effectiveness of consultation interventions but also examine the conditions under which they achieve the greatest benefits for families.

### 4.4. Limitations

A potential limitation of this review was the exclusion of publications on occupational therapy consultation that focused on issues related to parents themselves, without considering their child [50, 51]. Additionally, studies published before the period of the present review [52, 53], those in languages other than English [54, 55], and studies conducted in specific contexts, such as care units [56, 57] and school‐based environments [58–60], were excluded. These studies often involved consulting services provided in collaboration with teachers and other professionals in medical or educational settings. Furthermore, while studies that did not utilize occupational performance outcomes were excluded, it is important to note that this exclusion criterion did not result in the removal of any study solely based on the absence of performance‐based outcomes. The majority of excluded studies were removed for other reasons, such as being conducted in settings outside the scope of the review (e.g., school or care units). This ensures that the review still comprehensively reflects the impact of consultation on occupational performance in the relevant contexts.

Another methodological limitation concerns the data extraction process. Data extraction was conducted by a single author under the direct supervision and partial verification of two co‐authors, rather than by two independent reviewers. Although cross‐checking and consensus‐based verification of approximately 30% of the extracted data were applied to reduce potential inaccuracies, this procedure may still introduce a minor risk of selection or interpretation bias. Future scoping reviews are encouraged to employ dual independent extraction to further enhance methodological robustness.

## 5. Conclusion

This scoping review indicates that family‐centered consultation in occupational therapy can strengthen parental self‐efficacy and support children′s participation, yet the evidence remains limited and inconsistent across populations, contexts, and study designs. To maximize its impact, consultation must be implemented through culturally sensitive approaches and supported by therapist training that ensures family engagement and sustainability in natural environments. Future research should address the identified gaps by examining underrepresented diagnostic groups and age ranges, testing diverse delivery formats such as telehealth and group‐based models, and evaluating long‐term outcomes to provide robust evidence for practice and policy.

## Conflicts of Interest

The authors declare no conflicts of interest.

## Funding

No funding was received for this manuscript.

## Supporting information


**Supporting Information** Additional supporting information can be found online in the Supporting Information section. The supporting information associated with this article include additional appendices that provide detailed information about the methodological process of this scoping review. File S1: The PRISMA‐ScR checklist used for reporting. File S2: The complete PubMed search strategy, including all search terms and limits applied. These materials are available online to support transparency, reproducibility, and comprehensive understanding of the review process.

## Data Availability

The data that support the findings of this study are available from the corresponding author upon reasonable request.
