# Supplementary material for: Family‐Centered Occupational Therapy Consultation for Children Under 18 Years Old: A Scoping Review
Source: Occup Ther Int. 2026 Jan 8;2026:1184326. doi: 10.1155/oti/1184326 (PMC12780857; doi:10.1155/oti/1184326)
Supplement: Supplementary file 1 — Supporting Information Additional supporting information can be found online in the Supporting Information section. The supporting information associated with this article include additional appendices that provide detailed information about the methodological process of this scoping review. File S1: The PRISMA‐ScR checklist used for reporting. File S2: The complete PubMed search strategy, including all search terms and limits applied. These materials are available online to support transparency, reproducibility, and comprehensive understanding of the review process. [file OTI-2026-1184326-s001.zip › Supplementary 2.docx]

**Supplementary 2.** Detailed Search Strategy (PubMed)

**Database:** PubMed (searched April 15, 2025)

**Limits applied:** English language; Publication dates January 1, 2000 – April 30, 2025; Participants aged birth through 18 years old; Species: Humans

**Search strategy:**

(consult* OR collabor* OR "collaborative consulting")

AND

(performance OR participation OR "occupational therapy")

AND

(child* OR infan* OR toddler* OR adolescent OR youth)

AND

("family centered care" OR "parent involvement")

**Field tags used:** All fields

**Record identified:** 240
